# Sonographic features of adenomyosis correlated with clinical symptoms and intraoperative findings: a case–control study

**DOI:** 10.1007/s00404-022-06852-2

**Published:** 2023-03-11

**Authors:** Bashar Haj Hamoud, Mariz Kasoha, Martin Sillem, Erich-Franz Solomayer, Romina-Marina Sima, Liana Ples, Roxana Schwab, Gregor Leonhard Olmes

**Affiliations:** 1 Department of Obstetrics and Gynecology, Saarland University Hospital, Homburg, Germany; 2 Department of Obstetrics and Gynecology, “Carol Davila” University of Medicine and Pharmacy, “Sf. Ioan” Hospital-Bucur Maternity Bucharest, Bucharest, Romania; 3 Department of Gynecology and Obstetrics, University Medical Center Mainz, Langenbeckstraße 1, 55131 Mainz, Germany

**Keywords:** Adenomyosis, Sonographic, Hysteroscopy, Laparoscopy

## Abstract

**Purpose:**

Adenomyosis is a common disease of females during their reproductive age. As of today, histologic examination of the uterus after hysterectomy constitutes the gold standard for diagnosis. The aim of this study was to determine the validity of sonographic, hysteroscopic, and laparoscopic criteria for the diagnosis of the disease.

**Methods:**

This study included data collected from 50 women in the reproductive age of 18–45 years, who underwent a laparoscopic hysterectomy in the gynecology department of the Saarland University Hospital in Homburg between 2017 and 2018. The patients with adenomyosis were compared with a healthy control group.

**Results:**

We collected data of anamnesis, sonographic criteria, hysteroscopic criteria and laparoscopic criteria and compared it with the postoperative histological results. A total 25 patients were diagnosed with adenomyosis postoperatively. For each of these; at least three sonographic diagnostical criteria for adenomyosis were found compared with a maximum of two for the control group.

**Conclusion:**

This study demonstrated an association between pre- and intraoperative signs of adenomyosis. In this way, it shows a high diagnostic accuracy of the sonographic examination as a pre-operative diagnostic method of the adenomyosis.

## What does this study add to the clinical work

**Table:** 

Pre-operative ultrasonography is an accurate diagnostic method for adenomyosis. Intraoperatively the uteri of patients with adenomyosis present specific morphologic features. Combining preoperative with intraoperative findings can have important diagnostic implications for patients with adenomyosis.	

## Introduction

Adenomyosis, the presence of endometrial glands and stroma in the myometrium, constitutes a benign entity presenting with dysmenorrhea, pelvic pain, abnormal uterine bleeding, and/or infertility [1, 2]. Of note, adenomyosis and endometriosis are considered two different entities with very much different etiopathologies and treatment strategies, as pointed in the latest guidelines for endometriosis by the European Society of Human Reproduction and Embryology (ESHRE) [3].

Even though a series of mechanisms have been proposed, the etiology of the disease remains enigmatic [4, 5]. Currently, the most accepted theory is that of a disrupted endomyometrial junction resulting in direct invasion of endometrial cells from the basalis layer into the myometrium because of continuous tissue injury signals [4, 5].

Estimating the exact prevalence of adenomyosis is linked with significant limitations [6, 7]. For example, assessing the prevalence at hysterectomy (the diagnostic gold standard) is prone to selection bias [7]. Hysterectomy is performed in patients with complaints, which means that asymptomatic patients are excluded from prevalence estimation [7]. According to a recent review by Upson and colleagues, reported estimates range from 8 to 60%, depending on histologic criteria used by each study [7]. Furthermore, estimating the prevalence of adenomyosis based on diagnosis at hysterectomy increases the mean age of patients diagnosed with the disease, leaving younger women out of the estimation [7].

Regarding treatment, there is controversy regarding the optimal therapy for adenomyosis [8]. An important pillar of treatment is medical treatment with analgesics as well as hormonal therapies being available [9]. These include gonadotropin releasing hormone antagonists (GnRHa), levonogestrel-releasing intrauterine devices (LNG-IUD), dienogest, or ulipristal acetate [9]. Surgical uterus-sparing concepts have also developed in recent years and are being propagated for the treatment of women with infertility [10]. Given the high burden of the disease on the patients and health systems as well as a correlation with deep infiltrating endometriosis, which might require even more extensive surgery (thus increased morbidity), early non-invasive diagnosis is of vital importance [11, 12].

An important part of non-invasive diagnosis is ultrasound [13]. Sonography is becoming a first-line method for diagnosing adenomyosis with comparable diagnostic accuracy as the golden standard [13, 14]. Recently, an international consortium of expert sonographers proposed the use of standardized examination system for the sonographic examination of patients with adenomyosis [15]. These criteria include a series of diagnostic signs as well as standardized examination for diagnosis adenomyosis [15].

What remains uncaptured is the correlation between pre-operative findings, intraoperative signs, and histologic diagnosis of adenomyosis. The aim of this case–control study is to investigate this correlation and compare the presence of known signs indicative of adenomyosis in patients with histologically proven adenomyosis versus healthy controls.

## Materials and methods

### Setting and participants

We conducted a case–control study of patients with adenomyosis undergoing hysterectomy between January 2017 and December 2018 at the University Hospital of Saarland in Homburg, Germany. Eligibility criteria included known patients being treated at our endometriosis clinic of reproductive age, histologic diagnosis of adenomyosis after hysterectomy, and absence of leiomyomas. Patients should have been operated on hysteroscopic and laparoscopic once before hysterectomy. Exclusion criteria included current ongoing medical treatment, ongoing pregnancy, or incomplete clinical data. Patient selection was conducted after screening of electronic health records of the hospital. Patient data were extracted into a predefined Excel file.

### Clinical data

All patients completed a standardized clinical questionnaire, which included questions about dysmenorrhea, the pain severity in cases of dysmenorrhea classified using the visual analog scale (VAS) [16], dyspareunia, hypermenorrhea, dyschezia, dysuria (especially during or just before the period), hormone therapy, previous operations for endometriosis and pregnancy and parity.

### Ultrasonographic features

**Fig. 1:**
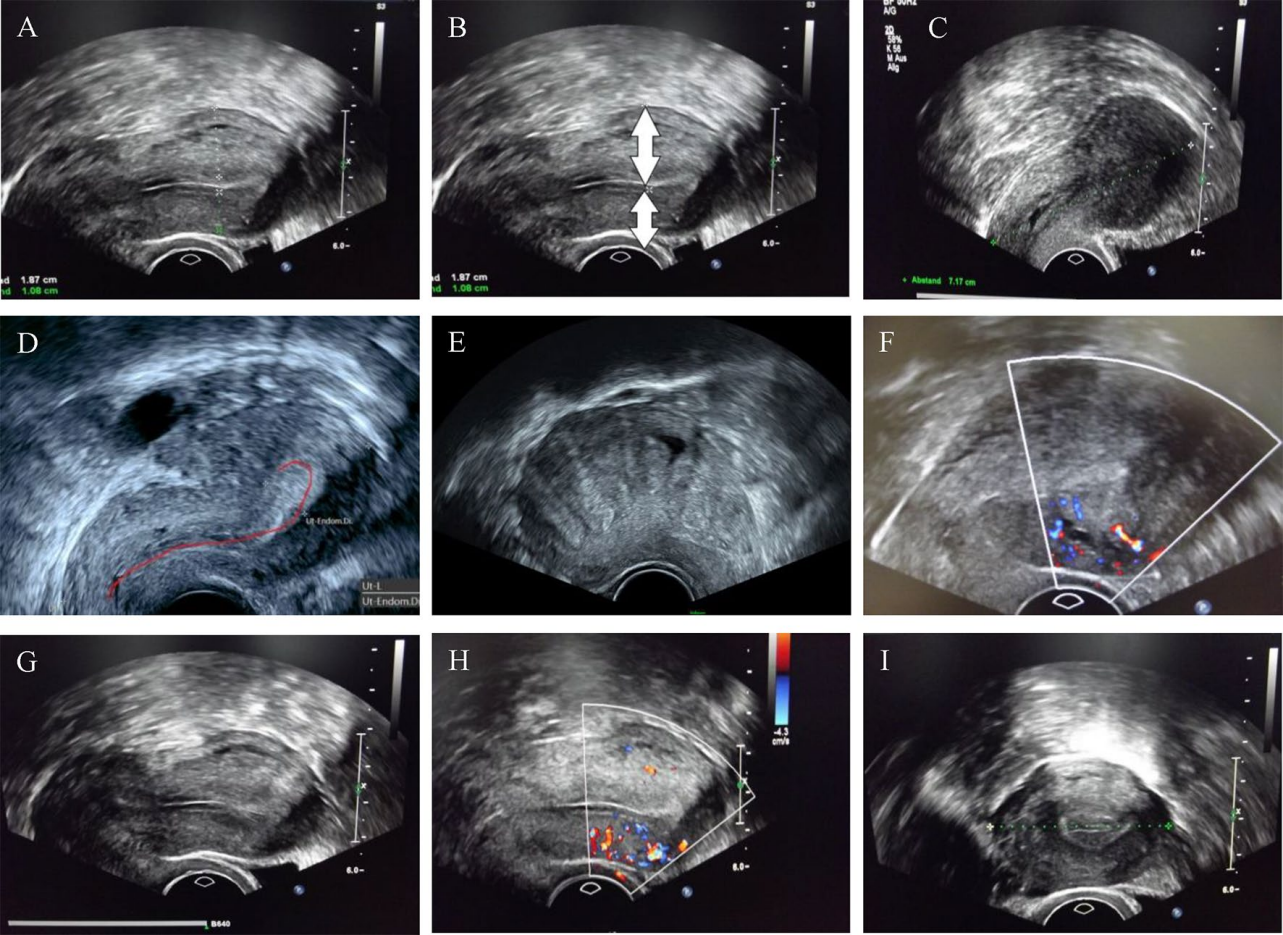
Adenomyosis-specific sonographic markers. **A** Asymmetric walls of uterus. **B** Asymmetric uterus walls. Marked for optimal visualization. **C** A blurred junctional zone of the endometrium and myometrium. **D** A question mark form of the uterus. **E** “Rainforest-Phenomen” of the uterus. **F** Intramural lacunae of the myometrium. **G** Heterogenic echogenicity of the myometrium. **H** Diffuse vascularisation of the myometrium. **I** Enlarged globular fundus uteri. All images were taken in our clinic

All patients were preoperatively examined in a systematic way from experienced gynecologic sonographers using Arietta 65, Hitachi. Eight features of adenomyosis were obtained: an anterior–posterior wall asymmetry of the uterus (Fig. 1A, B) [13], an irregular/blurred junctional zone (Fig. 1C) [13], a question mark shape of the uterus (Fig. 1D) [17], a “Rainforest Phenomenon” (Fig. 1E) [17, 18], intramural lacunae (Fig. 1F) [17, 18], a heterogeneous echogenicity of the myometrium (Fig. 1G) [17, 18], a diffuse vascularisation of the myometrium (Fig. 1H) [17, 18], and an enlarged globular fundus uteri (Fig. 1I) [17, 18].

### Intraoperative findings

**Fig. 2:**
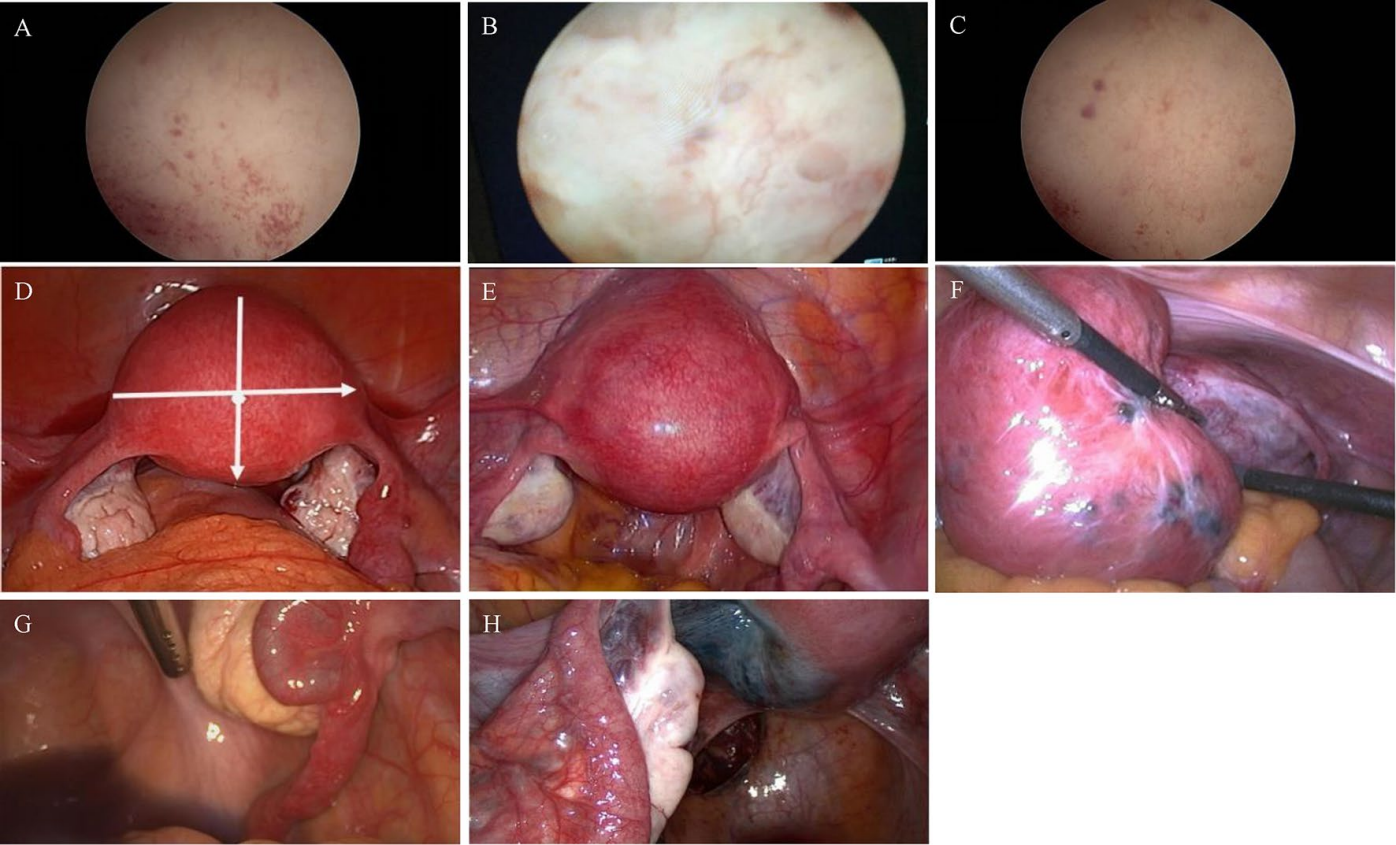
Hysteroscopic and laparoscopic criteria of endometriosis. **A** Strawberry sign in hysteroscopy. **B** Lacunar formation in the endometrium in hysteroscopy. **C** Hypervascularisation and cystic haemorrhagic lesions in the area of the endometrium in hysteroscopy. **D** Enlargement of the corpus uteri and a spherical shape of the uterus in laparoscopy. **E** A marbled serosa over the uterus in laparoscopy. **F** Brownish cysts on the uterine wall in laparoscopy. **G** The assessment of the tube permeability in laparoscopy. **H** The “Blue Sign” in laparoscopy. All images are taken in our clinic

To demonstrate possible correlations between sonographic criteria and intraoperative hysteroscopic and laparoscopic findings in the presence of adenomyosis, the following hysteroscopic and laparoscopic criteria were examined for their presence. The four hysteroscopic features were the “Strawberry Sign” (Fig. 2A), lacunar formations of the endometrium (Fig. 2B), hypervascularisation, and cystic haemorrhagic lesions in the area of the endometrium (Fig. 2C) [19]. Apart from hysteroscopic, five laparoscopic features were obtained; enlargement of the corpus uteri > 9 cm, a spherical shape of the uterus (Fig. 2D), a soft, doughy uterine wall, a marbled serosa over the uterus (Fig. 2E), and brownish cysts on the uterine wall (Fig. 2F) [20]. Additionally, we extracted information on the results of the tubal patency test (with methylene blue) from previous operations (Fig. 2H) [18]. The presence of the “Blue sign”, namely the acquisition of blue color mostly in the posterior wall of the uterus as a result of the altered myometrium and/or disrupted junctional zone seen in adenomyosis, was also extracted (Fig. 2G) [18].

For a better assessment of the endometrium, all operations in our department are carried out during the first half of the cycle. Endoscopic operations were conducted using conventional instruments described in the previous publications [21].

The patients with adenomyosis were compared with a control group of 25 age-matched patients. The patients in the control group were premenopausal women who had a hysterectomy for an indication other than adenomyosis or a myoma. These indications included cervical intraepithelial neoplasia, hysterectomy for gender dysphoria, and uterine prolapse. These patients were assessed for the same clinical, sonographical, and intraoperative criteria for adenomyosis as the endometriosis patients. The results between the groups were compared.

### Statistical analysis

After consultation with the local Institute for Medical Statistics, we refrained from a statistical analysis with the determination of statistical significance because of the monocentric character of the work. Instead, a descriptive analysis was carried out. The data were analyzed using “Microsoft Excel” program. After complete data extraction, the absolute and relative frequencies of each criterion in the different groups were determined. In this way, the absolute and relative frequencies of the criteria in relation to the parameters could be evaluated. The results are presented in tables and graphics.

## Results

A total of 25 patients with adenomyosis in the histological work-up after hysterectomy fulfilled the eligibility criteria and were enrolled in the study. Clinical characteristics are presented in Table 1. The median age of the patients was 30 years (Range: 18–45 years). Each of the 25 patients with a postoperative diagnosis of adenomyosis had previous surgery for endometriosis (hysteroscopy, laparoscopy with removal of endometriosis lesions, and chromopertubation).

**Table 1:** The distribution of symptoms and intraoperative signs in adenomyosis (*n*=25)

	Adenomyosis *n*=25
Dysmenorrhea	25 (100%) VAS range 7–10
Dyspareunia	20 (80%)
Hypermenorrhea	22 (88%)
Blue sign	
Present	10 (40%)
Absent	15 (60%)
Tubal permeability	
Both tubes permeable	7 (28%)
One tube permeable	15 (60%)
No tubes permeable	3 (12%)

### Sonographic features

The minimum sonographic portio-fundus (measured intraoperatively) distance was 7 cm and the maximum 12 cm. The median sonographic portio-fundus distance in adenomyosis was 9.5 cm. In the comparison group, the portio-fundus distance varied between 4 cm (minimum) and 8.5 cm (maximum). The median portio-fundus distance in the patients without adenomyosis was 6 cm.

The uterine width varied between 4 cm (minimum) and 7 cm (maximum) in adenomyosis. In the patients of the comparison group, the median uterine width was 3.5 cm. Twenty of the 25 patients with adenomyosis (80%) had diffuse vascularization of the myometrium on sonography, compared to only 7 of 25 patients (28%) in the comparison group. An anterior–posterior wall asymmetry of > 1/3 was present in 80% of the adenomyosis patients (20 of 25 patients). In the comparison group, only 5 out of 25 patients (20%) had anterior–posterior uterine asymmetry. Intramural lacunae were present in 15 of 25 (60%) patients. In women without adenomyosis, intramural lacunae were found in 5 of 25 cases (20%).

A blurred junctional zone was present in more than half of the patients with adenomyosis in the region of the myometrium (in 13 of 25 patients or 52%). In the comparison group, 3 out of 15 women (12%) were affected. A question mark form of the uterus was present in only about 1/3 of the patients (8 of 25 patients) with adenomyosis. In the control group, 2 out of 25 patients (8%) had a question mark form. An enlarged, spherical fundus was also detected in about 1/3 (8 out of 25 patients) of the adenomyosis patients, but only in 3 out of 25 patients (12%) of the comparison group.

The rainforest sign was detected preoperatively in 7 of 25 adenomyosis patients (28%) and in 2 patients (7%) without adenomyosis. A heterogeneous echogenicity of the myometrium was seen in 8 of 25 (32%) patients with adenomyosis and in 4 of 25 patients (16%) without adenomyosis. In all patients with histologically confirmed adenomyosis, at least three ultrasound criteria of adenomyosis could be detected, while in the control group, the maximum number of sonographic features demonstrated per patient was two.

### Hysteroscopic features

The strawberry sign was detected in 15 of the 25 patients with adenomyosis (60%) during hysteroscopy. Thirteen of 25 patients (52%) had hypervascularisation. Lacunae in the endometrium and cystic, haemorrhagic lesions of the endometrium were detected in 7 of 25 patients (28%). All patients with confirmed adenomyosis had at least two hysteroscopic features.

### Laparoscopic features

The minimum intraoperative probe length for adenomyosis was 7 cm. Fifteen patients had a probe length > 9 cm. In the comparison group, 16 of 25 patients (64%) had an intraoperative uterine probe length between 6 and 7 cm. The uterus was spherical in 22 of 25 (88%) adenomyosis patients. In the comparison group, three patients (12%) had a spherical shape of the uterus.

Thirteen out of 25 (52%) of the patients with adenomyosis had a doughy uterine wall intraoperatively. Of the patients in the comparison group, only 2 out of 25 patients (8%) had a doughy uterine wall.

A total of 7 out of 15 patients (28%) with adenomyosis had a marbled uterine surface intraoperatively. In contrast, 2 out of 25 patients (8%) without adenomyosis had a marbled uterine surface.

Only 3 out of 25 patients (12%) with adenomyosis had cysts on the uterine wall. Two out of 25 women without adenomyosis (8%) had uterine cysts.

### Tubal patency test

Of the 25 patients with adenomyosis, ten had a blue sign intraoperatively. In terms of tubal permeability, in seven (28%) both were patent, in fifteen only one was patent, and in three there was no tubal patency (Table 1).

All adenomyosis patients had at least two intraoperative features. Patients without adenomyosis had a maximum of one laparoscopic finding typical of adenomyosis.

## Discussion

This monocentric case–control study has been able to clarify the significance of clinical, sonographic, and operative criteria in the diagnosis of adenomyosis. Patients with adenomyosis present with at least three sonographic signs compared two in healthy controls. Regarding intraoperative signs, these were more common in patients diagnosed with adenomyosis compared with controls.

The significance of sonographic signs in adenomyosis has been studied by various working groups. Nevertheless, no superiority has been demonstrated for one specific sign. For example, Sun and colleagues demonstrated that subendometrial echogenic striae (equivalent to the rainforest sign) had the best sensitivity for diagnosis adenomyosis among various other sonographic markers [22]. A recent meta-analysis by Anders et al*.* demonstrated that myometrial heterogeneity was associated with highest sensitivity and a globular uterus with the highest specificity [17]. Of note, both parameters were increased when the question mark sign was incorporated in the analysis [17]. In a cohort of young women solely with adenomyosis, the asymmetrical myometrial wall morphology was the most common sonographic sign [23]. In our study, the validity of the sonographic parameters is underlined by the fact that all sonographic parameters were detected significantly less frequently in women of the comparison group.

An important advantage of our study was the completeness of the data as well as the systematic evaluation of patient history, ultrasonographic findings, and intraoperative findings. Structured reports and their role in the diagnosis of adenomyosis have been studied by Ribeiro da Silva et al*.* [24]*.* According to the researchers, structured ultrasound reports (as the ones used in our studies) are more effective in diagnosing adenomyosis compared with non-structured ones [24].

All patients in our study underwent standardized procedure by expert sonographers and laparoscopists. Furthermore, our study investigated signs obtained during hysteroscopy, laparoscopy, and chromopertubation (e.g., the blue sign), and correlated them with pre-operative findings and definitive histology.

Despite its strengths, the present study has methodological limitations. The strongest limiting factor is the small number of patients included in the study. Small number statistics and inference can be prone to limitations, as a few patients can cause a considerable effects in the data analysis as pointed out by Button and colleagues [25]. Therefore, in consultation with the local Institute for Medical Statistics, we did not determine the statistical significance and limited ourselves to a descriptive analysis. Furthermore, the data stem exclusively from one center, which might have implication for extrapolation. Finally, the retrospective nature of the study might pose limitations in terms of generalization in other cohorts.

In addition to sonography, magnetic resonance imaging can also be used in the non-invasive diagnosis of adenomyosis [26, 27]. Studies have demonstrated sensitivity of almost 90% making MRI comparable and sometimes more reliable than ultrasound (i.e., patients with increased body mass index) [26, 27]. Its comparability with ultrasound has already been studied [28]. The advantages of MRI are the low inter-examiner variability, the high sensitivity and specificity, and the almost unsurpassed soft-tissue contrast [26, 27]. However, due to its lower availability, longer examination time, and higher equipment and maintenance costs, MRI is far less suitable for widespread use than sonography under everyday conditions, especially in the outpatient setting [26, 27]. Future prospective studies incorporating MRI findings as well as ultrasonographic signs with intraoperative and histologic criteria could be more insightful.

## Conclusions

This study shows a high diagnostic accuracy of the sonographic examination as a pre-operative diagnostic method of the adenomyosis. Furthermore, it demonstrates an important association between intraoperative signs (both hysteroscopic and laparoscopic), histologic diagnosis, and pre-operative assessment. Taken together, these signs can play an important role in coining the diagnosis of adenomyosis.

## Data Availability

The dataset used and analyzed for th study is available from the corresponding author on reasonable request.
